# Prediction of miRNA-disease Associations using an Evolutionary Tuned Latent Semantic Analysis

**DOI:** 10.1038/s41598-017-10065-y

**Published:** 2017-09-05

**Authors:** Denis Pallez, Julien Gardès, Claude Pasquier

**Affiliations:** 1 Université Côte d’Azur, CNRS, I3S Sophia Antipolis, France; 2 BIOMANDA, 2720 Chemin St Bernard, Les Moulins I Batiment 4, 06220 Vallauris, France

## Abstract

MicroRNAs, small non-coding elements implied in gene regulation, are very interesting biomarkers for various diseases such as cancers. They represent potential prodigious biotechnologies for early diagnosis and gene therapies. However, experimental verification of microRNA-disease associations are time-consuming and costly, so that computational modeling is a proper solution. Previously, we designed MiRAI, a predictive method based on distributional semantics, to identify new associations between microRNA molecules and human diseases. Our preliminary results showed very good prediction scores compared to other available methods. However, MiRAI performances depend on numerous parameters that cannot be tuned manually. In this study, a parallel evolutionary algorithm is proposed for finding an optimal configuration of our predictive method. The automatically parametrized version of MiRAI achieved excellent performance. It highlighted new miRNA-disease associations, especially the potential implication of mir-188 and mir-795 in various diseases. In addition, our method allowed to detect several putative false associations contained in the reference database.

## Introduction

MicroRNAs (miRNAs) are a class of 19–24 nucleotides single-stranded non-coding RNAs that can regulate gene expression at the post-transcriptional level by binding with 3′ untranslated regions (UTRs) of the target mRNAs through base pairing. Every miRNA might regulate from a dozen to thousands of genes and one target gene could also be regulated by hundreds of miRNAs. These miRNA-mRNA interactions play critical roles in many physiological processes, such as development, apoptosis, differentiation and metabolism. miRNA dysregulations are also closely related to the development and progression of various human diseases, including cancer. Therefore, identifying new microRNAs associated with diseases contributes to a better understanding of pathogenicity mechanisms.

The state of knowledge in this field is still relatively limited at the current time. In addition, the cost of Research and Development (R&D) in “wet” laboratories to reach a new level of understanding can be a brake in scientific progression. To increase the chance of success and to focus biologists on promising ways, computational modeling is still a proper solution.

Because miRNAs act mainly by targeting mRNAs for cleavage or translational repression^1^, the first proposed methods inferred miRNA-disease associations from the known associations between targeted mRNAs and diseases. Subsequently, a significant number of methods were presented that took into account various data sources (disease phenotypic similarity, miRNA functional similarity, miRNA family). Surveys of the existing computational approaches, their performance and their limitations can be found in Zou *et al*.^2^ and Zeng *et al*.^3^.

Several recent works combined multiple sources of information to build integrated methods capable of achieving an excellent accuracy.

Liu *et al*.^4^ proposed a method that combines a disease similarity network and a miRNA similarity network to build an heterogeneous network explored by a random walk. The authors built those networks by integrating multiple data sources. The disease similarity is composed of disease semantic similarity and disease functional similarity, and the miRNA similarity is calculated using the miRNA-target gene and miRNA-lncRNA (long non-coding RNA) associations.

On a similar network, built by integrating different sources, Yu *et al*.^5^ used a combinatorial prioritization algorithm^6^ to prioritize disease-microRNA associations. This is achieved by computing, for each combination of disease-microRNA, an association score that is obtained by maximizing network information flow. This method makes it possible to infer new associations for a disease or a miRNA, even in the absence of known associations. Yu *et al*.^5^ also investigated an ensemble-based method that obtains very high performance.

Gu *et al*.^7^ combined miRNA functional similarities, miRNA family information and miRNA-disease associations to build a miRNA-miRNA similarity network (the miRNA space). Similarly, they used disease semantic similarity and miRNA-disease associations to build a disease-disease similarity network (the disease space). The score of an association between miRNA *m* and disease *d* depends on the spatial similarity between the miRNAs associated to *d* and the connexions of *m* in the miRNA space and, conversely, the score depends also on the spatial similarity between the diseases associated to *m* and the connexions of *d* in the disease space.

You *et al*.^8^ proposed a prediction model that integrates known human miRNA-disease associations, miRNA functional similarity and disease semantic similarity. Based on the assumption that miRNAs with more functional similarity tend to be associated with similar diseases, the authors use Gaussian interaction profile kernel^9^ for calculating the similarities network between diseases. The method constructs a heterogeneous graph consisting of three interlinked sub-graphs (i.e., miRNA-miRNA similarity network, disease-disease similarity network and miRNA-disease association network) and further adopts depth-first search algorithm to infer potential new miRNA-disease associations. In addition to achieving excellent performance, this method allows to predict new associations for diseases with no known associated miRNAs (or for miRNAs with no known associated diseases).

Pasquier *et al*.^10^ made the assumption that information attached to miRNAs and diseases can be revealed by distributional semantics. The approach represented distributional information on miRNAs and diseases in a high-dimensional vector space and defined associations between miRNAs and diseases in terms of vector similarity. Cross validations performed on a dataset of known miRNA-disease associations demonstrated the excellent performance of the method and its ability to discover new disease-miRNA associations as well as to identify putative false associations reported in databases.

The problem with this approach is the need to define the set of control parameter values whose evaluation of each combination is impossible. In the work of Pasquier *et al*.^10^, parameter values were found manually by the authors from many trial-error iterations. In this paper, we focus on the use of Evolutionary Algorithms (EA) to determine, in a reasonable time, a satisfactory tuning without having to evaluate all the possible configurations. In order to reduce computational time, configurations are evaluated in parallel on a computation-grid. To determine which configuration is needed for a real evaluation on the grid, a surrogate model is employed during the EA process. This strategy allowed us to significantly increase the performance of the predictions.

## Methods

### microRNA-disease association prediction method (MiRAI)

In a previous work, we developed a method, called *MiRAI*, that uses distributional semantics to reveal new information attached to miRNAs and diseases^10^. Our basic approach represents distributional information on miRNAs in a high-dimensional vector space^11^ and defines the associations between miRNAs and diseases in terms of vector similarities. The vector space model is an algebraic model for representing objects as vectors. Our vector space model represents miRNAs as vectors in a *d*-dimensional space, where *d* is the number of unique attributes that characterize a miRNA. The *d* components of each miRNA vector are assigned with a number (a weight) that quantifies the importance of an attribute in the modelized miRNA.

We used Latent Semantic Analysis (LSA)^12^ to process vectors from the vector space model we created. Singular Value Decomposition (SVD) is used for reducing the dimension of the original matrix while preserving the similarity structure. In SVD, a rectangular matrix is decomposed into the product of three other matrices. One of the resulting matrices describes the original row entities as vectors of arbitrary size, another matrix describes the original column entities as vectors of the same size. Rows and columns are thus the components of a same dimensional space. They can be compared by taking the cosine of the angle between their corresponding vectors. Values close to 1 represent very similar data while values close to 0 represent very dissimilar data.

Each miRNA can be characterized by several kinds of data: known associated diseases, target mRNAs, family, proximity to neighbor miRNAs, abstracts of associated papers and other descriptions in plain text format. For textual data, there exists many ways to calculate weights and numerous studies were dedicated to the finding of an efficient weighting scheme^13^. For numerical or categorical data, nothing like this exists. One needs to try different weighting schemes for each kind of data to evaluate their pertinence in the frame of LSA. However, the evaluation of the weighting schemes cannot be performed one after the other. All combinations of different possible strategies for calculating the weight of each piece of data should be considered.

**Figure 1:**
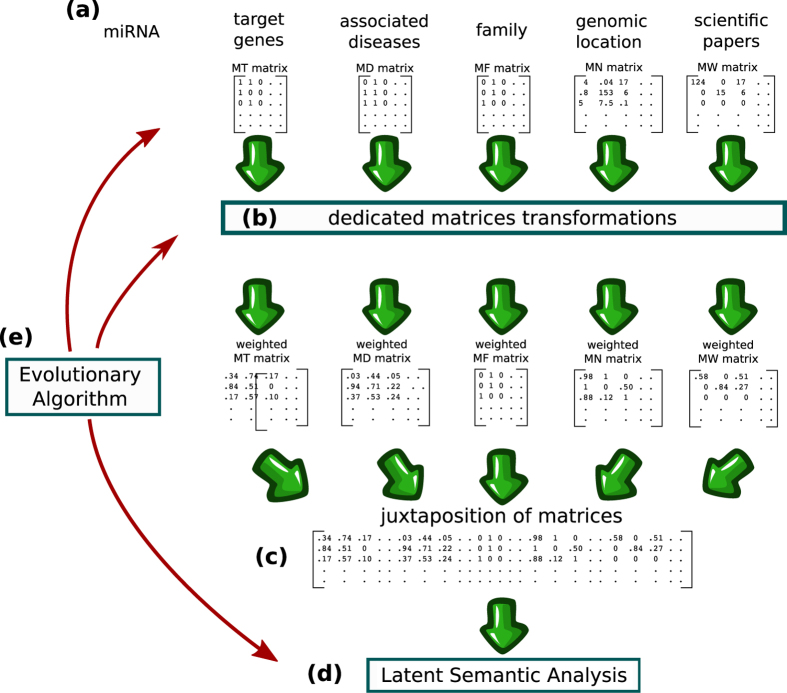
Illustration of the method. (**a**) miRNAs are characterized by several kinds of data that are stored in distinct matrices. (**b**) Each matrix is processed by a dedicated method for transforming it into a weighted matrix where the strength of an association between a miRNA and a characteristic is represented by a float number. (**c**) Concatenation of matrices (**d**) similarities and dissimilarities between miRNAs and diseases are highlighted by LSA. (**e**) Evolutionary computation is used for selecting the data sources to use, for tuning the matrices transformations, for determining the size of the latent space and for choosing to whether expand or not the terms used for LSA queries.

Roughly speaking, as depicted in Fig. 1, we have four different sources of data that we can choose to use or not (associated diseases being a mandatory source). In fact, using the maximum amount of data is not necessarily the best option, as we shall see later. For miRNA-target data, we have the choice of whether to use the raw values or to transform these values by using, for instance, a recommendation algorithm. For miRNA-disease associations, we have several ways to compute the similarities between diseases. For genomic location, we can imagine several strategies to weight the likelihood of a coexpression pattern. For plain text data, we can use the raw counts of words or use the popular Term Frequency–Inverse Document Frequency weighting scheme (TF-IDF)^11^.

We can also choose between several dimensions from the reduced space. In total, we enumerated 35 different alternatives that influence the behavior of the algorithm. For each parameter, we have the choice between two values for a total of 2^35^, or 5 × 10^16^ different configurations. Depending on the data used, the resulting matrix can have tens of thousands of columns and the training of the model can last up to four hours. Even by automating the process with brute force search, the combinatorial explosion makes this impractical. For this kind of problems, where the enumeration of all the solutions is not possible, some meta-heuristics algorithms, such as evolutionary computation, can be applied.

### Determination of parameters

As described previously, each miRNA is associated with several kinds of data that are stored in distinct matrices (Fig. 1a). In all matrices, rows represent miRNAs and columns represent distinct characteristics. In miRNA-target associations matrix (MT matrix), columns represent genes and the entry in the *i*-th row and *j*-th column *mt*_*ij*_ is equal to 1 if miRNA at position *i* targets the gene at position *j*, 0 otherwise. In miRNA-disease associations matrix (MD matrix), columns represent diseases and *md*_*ij*_ is equal to 1 if the association between miRNA at position *i* and disease at position *j* has been reported, 0 otherwise. In miRNA-family associations matrix (MF matrix), columns represent miRNA families and *MF*_*ij*_ is equal to 1 if the miRNA at position *i* belongs to family at position *j*, 0 otherwise. In miRNA-neighbor associations matrix (MN matrix), columns represent miRNAs and *mn*_*ij*_ is equal to the genomic distance between the miRNA at row position *i* and the miRNA at column position *j*. In miRNA-word associations matrix (MW matrix), columns represent words and *mw*_*ij*_ is equal to the number of times the word at position *j* is used in plain text descriptions of the miRNA at position *i*. Concerning the sources of the plain text descriptions, we can opt to use the abstract of articles associated with miRNAs in PubMed (by performing searches with the name of the miRNAs), the abstracts referenced in MiRBase, the miRNA description fields in MiRBase or any combination of these sources.

Using information related to disease associations is mandatory but other data may or may not be used. So we have a total of 6 individual choices, depending on whether the corresponding data are used or not (Table 1a). If we elect to use the association between miRNAs and their targets, we can choose whether to use the raw associations stored in databases or to apply a method modulating the weight of the associations such as, for example the network based inference method described by Zhou *et al*.^14^ (Table 1b).

**Table 1:** Description and encoding of parameters.

Id	Description of the parameters	Type	Binary encoding
a	Inclusion of data sources		
	Use of target names	1 binary	bit 1
	Use of family data	1 binary	bit 2
	Use the proximity with neighbor miRNAs	1 binary	bit 3
	Use the abstract of associated PubMed papers	1 binary	bit 4
	Use the abstract of associated MiRBase papers	1 binary	bit 5
	Use the description of the miRNA in MiRBase	1 binary	bit 6
b	Transformation of data sources		
	Applying NBI on miRNA-target links	1 binary	bit 7
	Applying TF-IDF on PubMed abstracts	1 binary	bit 8
	Applying TF-IDF on MiRBase abstracts	1 binary	bit 9
	Applying TF-IDF onMiRBase descriptions	1 binary	bit 10
	Inference of subsumed diseases in matrix	1 binary	bit 11
	Discretization of disease similarities	x floats	bits [12–30]
c	Dimension of reduced space	1 integer	bits [31–34]
d	Inference of subsumed disease in the query	1 binary	bit 35

Concerning miRNA-disease associations, we can decide whether to use or not similarities between diseases as explained in Pasquier *et al*.^10^. However, instead of using an unique measure *m* ∈ [0, 1] reflecting the weight of the association between a miRNA and a disease, we decided to discretize this measure to obtain several indicators, each of them indicating whether the weight of the association is above a given cutoff or not. For example, by using 1/3 and 2/3 as cutoffs, one obtains three overlapping bins that associate *m* ∈ [0, 1/3] with the indicator no_assoc, *m* ∈ [1/3, 1] with the indicator significant_assoc and *m* ∈ [2/3, 1] with the indicator high_assoc. With this mapping, a similarity measure of 0.8 will be associated with the annotations moderate_assoc and high_assoc. The setting consists in choosing the number of cutoffs to use and selecting their values (Table 1b).

We also have to decide if we want to infer diseases. Inferring diseases means that if a miRNAs is associated with a term, then it is also associated with all the subsumed terms. For example, from the association of a miRNA with “Colonic Neoplasms”, we can infer that the miRNA is also associated with “Colorectal Neoplasms” because in the MeSH hierarchy, the term “Colonic Neoplasms” is subsumed by “Colorectal Neoplasms”. The inference of the diseases can take place in the matrix, before computing the disease similarities (Table 1b) or during the query (Table 1d). For the latter, the query is expanded with all the terms that are subsumed.

The proximity with other miRNAs is an important factor, as noted by several authors^15, 16^. If such information is taken into account, a weighting scheme is considered allowing each entry in the matrix to correspond to a value indicating the likelihood of a coexpression pattern^10^. If plain text data are used, we can decide whether to use or not a weighting scheme. Among the numerous existing weighting schemes, we stick to the popular TF-IDF weighting scheme^11^, which involves multiplying the Inverse Document Frequency measure by a Term Frequency measure (Table 1b). If we elect to use the associations between miRNAs and families, we do not apply any weighting scheme as every miRNA is associated with only one family.

The last parameter that has influence on the MiRAI method is the dimension of the reduced space. As summarized in Table 1, the MiRAI method is controlled by 12 binary parameters, a mix of real numbers (the discretization cutoffs) and 1 integer (the dimension of the reduced space). In the present work, the goal is to find the best combination parameter values for prediction. So, all previous parameters are gathered in an unique vector. In order to avoid dealing with different parameter types (binary, real or integer) and to simplify the optimization technique adaptation, we decided to convert all of them in the binary space. Indeed, only integer and real parameters are converted using respectively Gray coding and cutoffs.

Concerning the cutoffs, we decided to allow a maximum of 19 cutoffs and to pre-determine their value from 0.05 to 0.95 using a step of 0.05. This is represented by 19 binary values, each one corresponding to a cutoff value. The binary value is 1 when the cutoff is used, 0 otherwise (Table 1c). We encoded the dimension of the latent space on four bits to encompass all dimensions between 50 and 800 with a step of 50. The number *d* stored in the vector is encoded with reflected binary code (RBC). The dimension is obtained with *dim* = 50(*d* + 1) (Table 1c). Ultimately, each control parameter set of MiRAI is encoded by a binary vector of 35 bits as detailed in Table 1.

### Tuning MiRAI with Surrogate model Assisted EA

The accuracy of the method is measured by computing the Area Under the ROC Curve (AUC) (an AUC of 1 reflects perfect classification and an AUC of 0.5 indicates random classification)^17^. This section describes the parametrization of MiRAI using a surrogate model assisted evolutionary algorithm for maximizing the AUC.

#### Evolutionary Algorithms

EAs are nature inspired and stochastic algorithms that mimic Darwin theory for problem optimization^18^. Given a problem *P* to be maximized (resp. minimized), the goal is to find a solution *x* ^*^ such as *f*(*x* ^*^) = *max* (resp. *min*) {*f*(*y*)/*y* ∈ *S*} with *S* the set of all the possible solutions of *P*, $f:S\to {\mathbb{R}}$ is called *objective* function or *fitness* function and indicates the quality of the solution *y* for the problem *P*.

Many variants of EA are proposed in the literature (Genetic Algorithm, Evolution Strategies, Genetic Programming, Differential Evolution …) but they all share a common structure: a set of candidate solutions {*x*_*i*_ ∈ *S*}_0<*i*≤*n*_ for the problem *P*, called *population*, is created and most often initialized randomly. Then, each solution of the population, called *individual*, is evaluated using the fitness function *f*. Based on the fitness value of each individual, a selection operator is applied for choosing individuals that are allowed to create offspring. The latter are generated using genetic operators like crossover or mutation, each applied with a given probability. Such operators introduce variability in the solution space allowing to escape from local optima. Offspring is in turn evaluated using *f*. As the population size is considered constant, a replacement operator is applied for choosing which individual among parents plus offspring are kept for the next iteration. A single iteration is called a *generation* and is repeated until a given criterion is met. This criterion can be a constraint on the elapsed time, the number of function evaluations, the maximum number of generations without improvement, etc. A very good overview of EA has been proposed by Bartz-Beielstein *et al*.^19^ where they introduced many fundamental sub-domains such as multiple objectives, dynamic, noisy or expensive optimization problems such as the one we are facing in this article.

EAs are preferred to deterministic optimizations when |*S*| is huge to the extent that they allow to find promising solutions within a reasonable timeframe. They can also be used when *f* is non-differentiable or non-continuous. As EAs deal with a set of individuals, they can naturally be parallelized as discussed in a next section.

#### Discrete Differential Evolution

In a serie of experiments, Differential Evolution (DE), initially proposed by Storn and Price^20^, has proven to be more effective than some other EAs (The reader can refer to http://www1.icsi.berkeley.edu/~storn/code.html for source code on DE). It works as follows: for each individual in the population called *target* vector and formalized as ${x}_{i}^{t}$, a *mutant* vector ${\mu }_{i}^{t}$ associated to ${x}_{i}^{t}$ is first generated by adding the weighted difference between two randomly chosen vectors (*parameter* vectors ${x}_{{i}_{2}}^{t}$ and ${x}_{{i}_{3}}^{t}$) to a third chosen vector (*base* vector ${x}_{{i}_{1}}^{t}$) using Eq. 1:

1 $${\mu }_{ij}^{t}={x}_{{i}_{1}j}^{t}+F\cdot ({x}_{{i}_{2}j}^{t}-{x}_{{i}_{3}j}^{t})$$

where *i* ≠ *i*_1_ ≠ *i*_2_ ≠ *i*_3_; *i*_1_, *i*_2_ and *i*_3_ are randomly and uniformly chosen between 1 and the population size *λ*; $F\in {{\mathbb{R}}}^{+}$ is a scaling factor, controlling the amplification of the differential variation and ${x}_{ij}^{t}$ represents the *j*–th gene of the *i*–th individual in the population at generation *t*. Secondly, one child, called the *trial* vector ${x}_{i}^{t+1}$, is obtained by crossing the mutant vector ${\mu }_{i}^{t}$ and the target vector ${x}_{i}^{t}$ using Eq. 2:

2 $${x}_{ij}^{t+1}=\{\begin{array}{ll}{\mu }_{ij}^{t} & {\rm{if}}\,(rand\le CR)\,{\rm{or}}\,j=rand(i)\\ {x}_{ij}^{t} & {\rm{otherwise}}\end{array}$$

where *CR* is the crossover probability ranged in (0, 1). *rand* is a random value uniformly distributed within [0, 1); *rand*(*i*) is a random integer ranging between 1 and *N* where *N* is the number of individuals. Finally, the target vector is replaced with the best of either the trial or the target vector. As the initial DE operates in a continuous space, which means that ${x}_{i}^{t}$ are float-valued vectors, Wang *et al*.^21^ proposed a modified binary version of DE, called MBDE for tackling binary-coded optimization problems. MBDE keeps same strategy as initial DE but introduces a probability estimation operator $P({x}_{ij}^{t})$ defined in Eq. 3 for defining a probability of vector ${x}_{ij}^{t}$:

3 $$P({x}_{ij}^{t})=\frac{1}{1+{e}^{-\frac{2\cdot b\cdot [{x}_{{i}_{1}j}^{t}+F\cdot ({x}_{{i}_{2}j}^{t}-{x}_{{i}_{3}j}^{t})-0.5]}{1+2\cdot F}}}$$

where $b\in {{\mathbb{R}}}^{+}$ (*b* = 6 is suggested by the authors). Then, this probability is used for defining the mutant vector in the binary space according to Eq. 4 in place of Eq. 1.

4 $${\mu }_{ij}^{t}=\{\begin{array}{ll}1 & {\rm{if}}\,rand\le P({x}_{ij}^{t})\\ 0 & {\rm{otherwise}}\end{array}$$

This new adaptation of DE in the binary space is well suited for our problem but does not take into account a cost evaluation of MiRAI configurations.

#### Surrogate model Assisted Evolutionary Algorithm

As mentioned earlier, evaluating one configuration of MiRAI framework may take up to four hours of computation on a single core, resulting to a *time*-*consuming* or *expensive* fitness function. Using only a parallelization strategy alone would not be sufficient in this case as EA needs many function evaluations to reach an acceptable solution. Various approaches^22–25^ are proposed to reduce the computational cost by exploiting knowledge of past evaluations and are mainly based on meta-modeling. The idea is to learn a new model, called *surrogate* in the sequel, that approximates the expensive real fitness function. Therefore, the EA is hybridized with a learned model and is called Surrogate model Assisted Evolutionary Algorithm (SAEA)^24^. There exists two main ways for hybridization. The first one is *evolution control* where a *controlled* number of individuals are evaluated with the real fitness function while others are evaluated with the model. In *individual*-*based* evolution control, as indicated by its name, only a certain rate of individuals in each population is evaluated using the real fitness function. In *generation*-*based* evolution control, all individuals of the population are evaluated using either the real fitness function or the surrogate function. The second way of hybridization is to use EA for optimizing the model. The resulting optima are then re-evaluated on the real fitness function, in turn used for updating the *surrogate* model.

#### Gaussian Process surrogate model

Buche *et al*.^24^ suggest using Gaussian Process (GP or *Krigging* ^26^) as a surrogate model because it has the following properties: (1) it can approximate any function as Artificial Neural Network (ANN) does even with discontinuities or multi-modality, (2) it can predict the mean and the standard deviation of the fitness value of any new individual, (3) it has a very small number of hyper-parameters compared with ANN which can be set either by the user or by an optimizer. A drawback of such model is its computational cost which is in ${\mathscr{O}}({n}^{3})$ for the learning of thereof, in ${\mathscr{O}}({n}^{2})$ for predicting the standard deviation and in ${\mathscr{O}}(n)$ for predicting the mean of the fitness value of a new individual. However, the time required for learning the surrogate model can be considered insignificant compared with the time required for evaluating MiRAI configurations.

Given *m* expensive and potentially noisy evaluations of a computational experiment $Y={\{{y}_{i}\in {\mathbb{R}}\}}_{0\le i\le m}$ and their *m* corresponding input configurations $X={\{{x}_{i}\in {{\mathbb{R}}}^{d}\}}_{0\le i\le m}$, we want to estimate value of *y* = *f*(*x*) at a new untested configuration, ${x}_{k}\notin X$. Rather than claiming *f*(*x*) relates to some specific models (linear, cubic, quadratic…) and in order to create a meta-model of the unknown *f* function, GP makes no assumption on the smoothness of *f* but assumes that *f*(*x*) is a Gaussian function represented by *N*(*μ*, *σ* ^2^) at any point *x* where the mean and the standard deviation are two constants independent of *x*. For any *x*, *f*(*x*) is a sample of *μ* + *ε*(*x*), where $\varepsilon (x)\sim N\mathrm{(0},{\sigma }^{2})$. Compared to other modeling techniques, a spatial correlation *sc* between the output values is assumed to be stationary and depends on input values. It is expressed in Eq. 5 as:

5 $$\forall x,x^{\prime} \in {{\mathbb{R}}}^{d},sc(f(x),f(x^{\prime} ))\equiv sc(x,x^{\prime} )\equiv sc(x-x^{\prime} )=exp(-\sum _{i=1}^{m}\,{\theta }_{i}{|{x}_{i}-{x}_{i}^{^{\prime} }|}^{{p}_{i}})$$

where unknown parameters *θ*_*i*_ > 0 indicate the importance of *x*_*i*_ on *f*(*x*) and 1 ≤ *p*_*i*_ ≤ 2 the smoothness of *f*(*x*). As mentioned by Buche *et al*.^24^, {*θ*_*i*_, *p*_*i*_}_0≤*i*≤*m*_ are GP hyperparameters and they can either be set by the user or optimized by a maximum likelihood approach^22, 26^. The interest of using GP lies in its ability to predict a function value with its corresponding confidence interval *ξ* and without much additional computational cost. As explained by Emmerich *et al*.^22^ and Buche *et al*.^24^, Lewis *et al*.^27^ introduced a *merit* function *f*_*m*_ (Eq. 6) in place of the predictive function $\widehat{f}$ of the GP surrogate model in order to balance exploration of unexplored area of search space and exploitation of optimal solutions of $\widehat{f}$:

6 $$\forall x\in {{\mathbb{R}}}^{d},\quad {f}_{m}(x)=\widehat{f}(x)-\omega \xi (x),\quad \omega \in [0,3]$$

where a common suggested choice is *ω* = 2 for a minimization problem. Since we are dealing with a maximization problem, *ω* must be negative.

#### Distribution of real MiRAI configurations evaluation on a grid

As seen in the previous section, surrogate model is used for identifying promising areas of the search space and for largely minimizing computational costs. However, surrogate models must be learned or updated from real evaluations which can be done in parallel. So, in this paper, we want to leverage the advantages of GP surrogate model combined with the advantages of evaluating expensive real fitness function in parallel. A generation-based evolution control (SAEA) is then used for its simplicity. When using the real and expensive fitness function, each evaluation is performed in parallel over a distributed cluster. In theory, this means that the number of cores should be equal to the population size. But it is not mandatory: since in our case the time required for evaluating one configuration varies between two configurations, we decided to have fewer cores than the population size. On the opposite, surrogate model learning, fitness evaluations and other steps of the EA are applied sequentially on the same processor as the computational time is negligible compared with population evaluation. As in classical EA, number of generations to be evolved should be specified, parameter *μ* is introduced for representing number of generations using the surrogate model. Once the EA has identified optimum in the surrogate model, each individual of the population is evaluated using the real fitness function and the surrogate model is updated from previous real evaluations. In this way, the proposed algorithm is adopting a classical generational *master*-*slave* EA where each job is sent over the network using Scoop framework^28^. According to all previous discussions and adaptations, the algorithm used in this paper for tuning MiRAI framework is described in Algorithm 1 and is called Parallel GP model Assisted Binary Differential Evolution (PGPABDE).

**Algorithm 1:**
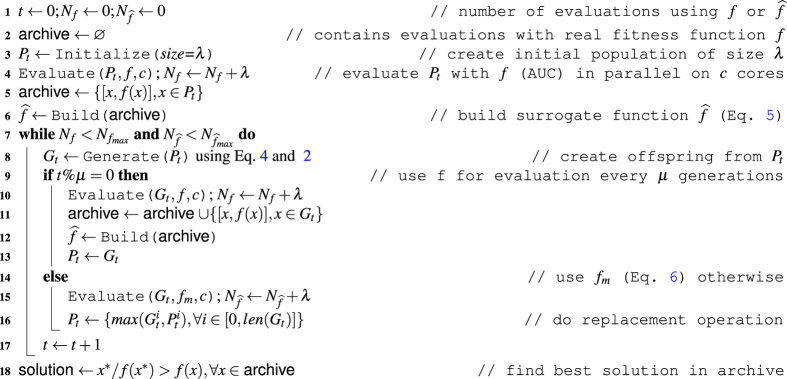
Parallel Gaussian Process model Assisted Binary Differential Evolution (PGPABDE).

In order to see the interest of surrogate model assisted EA, we compare the results of our algorithm (PGPABDE) with a modified version of thereof where no surrogate model is used (only real and expensive evaluations are used), called Parallel Binary Differential Evolution (PBDE). In that case, the number of surrogate generations *μ* is set to 0. Both algorithms were executed 9 times so as to obtain a fair comparison. The experimentations took place on the Interactive Computation Center of Nice Sophia-Antipolis University (Cluster for Education and Research of University Nice Sophia-Antipolis). Each of them were deployed on 3 nodes (48 cores) dealing with 50 individuals in the EA population over *t* = 50 generations. For accelerating the computation time, the 9 runs were divided into 3 parallel tasks. Finally, the experimentations used 18 nodes (288 cores) during approximately 11 days.

## Results and Discussion

### MiRAI configured with the evolutionary algorithm

**Figure 2:**
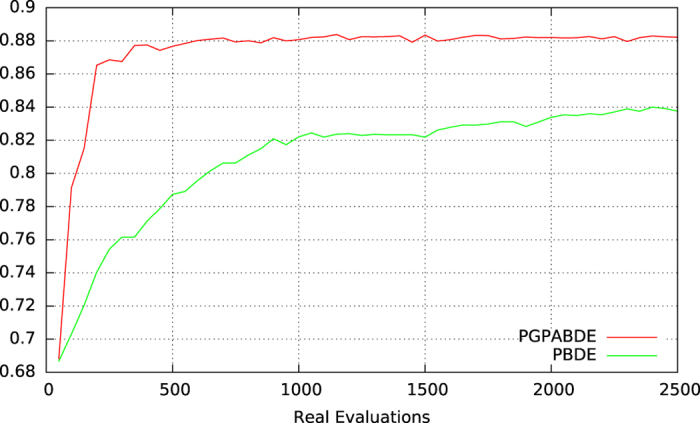
Average population fitness during MiRAI optimization (average on 9 independent runs).

**Figure 3:**
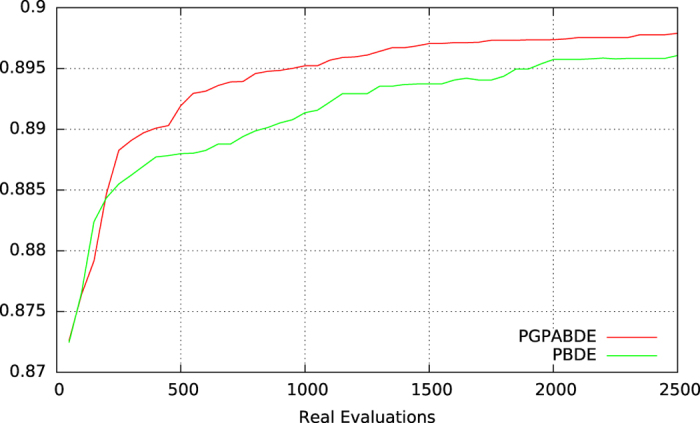
Best population fitness during MiRAI optimization (average on 9 independent runs).

MiRAI configuration has been optimized using algorithm 1 with parameter values described in Table 2. Results from experiments are averaged from all the independent runs and are depicted on the Supplementary Figs S1 and S2 which present the best, average and worst population fitness evolution for each experiment using PGPABDE and PBDE respectively. EA generation is traditionally considered as the time line for showing EA’s behavior. But, when considering expensive fitness function, the number of evaluations is preferred. It is also preferred for comparing both experiments. We can see that initial EA population is correctly balanced between good (around 0.87 in average) and bad (around 0.49) configurations. It is also worth noting that EA initialization was pretty good since best individuals in earlier steps are close to the optima found in average. It can be observed that the whole population of PBDE algorithm is converging more slowly than the PGPABDE algorithm but towards a similar optima (around 0.9 in average). When considering computational time (not depicted on figures), the average time used for training and evaluating 50 MiRAI configurations over one generation is about 110 minutes. In average, the 100 surrogate generations made after each real evaluation took 7 minutes (including the time for outputting logs). Figure 2 shows the average fitness of EA population during real evaluations. PGPABDE rapidly converges towards a score of 0.88 in average and stagnates around this optima after only 500 real evaluations while PBDE painfully reached 0.84 in average with 2500 real evaluations. We can see that using a surrogate model allows to reach better configurations with less real evaluations. When considering the best individual of EA population represented in Fig. 3, a score of 0.895 is reached with 1000 real evaluations using PGPABDE whereas it is necessary to wait for almost 2000 real evaluations using PBDE. Among all 9 runs, the best MiRAI configuration reaches the score of 0.903651231 after 1750 real evaluations using PGPABDE. On the opposite, the best configuration using PBDE was 0.899642612 after 2000 real evaluations.

**Table 2:** Parameter values of algorithm 1.

surrogate model exploration/exploitation	*ω* = −2
population size	*λ* = 50
number of cores	*c* = 48
max. real evaluations	*t* = 50, *N*_*f*_ = *t* × *λ*
max. surrogate evaluations	*μ* = 100, ${N}_{\widehat{f}}=\mu \times \lambda$
scaling factor	*F* = 0.8
crossover rate	*CR* = 0.8
individual length’s	*N* = 35
probability estimation operator	*b* = 6

An analysis of the best individual, i.e. the best MiRAI parametrization, found by PGPABDE algorithm reveals that, in addition to disease associations, only data relative to family, neighbor miRNAs and target genes are used (bits 1, 2, 3 of the encoding vector described in Table 1). Interestingly, none of the plain text sources of data were used. The encoding vector indicates that data relative to the associations with target genes have to be weighted with the Network Based Inference method (bit 7 described in Table 1). Diseases associated with miRNAs are first inferred from the data (bit 11 described in Table 1). Then, a similarity measure is performed between diseases and 3 cutoffs are used to discretize the value obtained. The optimal cutoffs encoded into the best individual are the following: 0.05, 0.25 and 0.65. Concerning the dimension of the reduced space, the EA confirmed the optimal value of 400 that was previously used with MiRAI. Eventually, the searched disease is extended with all the subsumed diseases before querying the latent space (bit 35 described in Table 1).

### Evaluation of prediction performance

To evaluate the ability of our method to predict disease-miRNA associations, a five-fold cross-validation is performed. For a specific disease *d*, the dataset is randomly partitioned into five equal-sized subsets. Four of five subsets are used to create the latent space, while the omitted subset is retained for querying and testing the model. During the latter test, all associations between miRNAs and *d* are removed before the update of the *MD* matrix with similarity data. The cross-validation process is then repeated five times, with each of the five subsets used exactly once as the validation data.

The latent space is then queried for the disease *d* to obtain a ranked list of miRNAs. The higher the miRNAs associated with *d* are ranked, the better the performance is.

The MiRAI software tuned with PGPABDE was tested on the 83 human diseases stored in the human miRNA-disease database (HMDD)^29^, that are associated with at least 20 miRNAs. The average AUC value obtained is 0.897 with a minimum of 0.713 for Lupus Vulgaris and a maximum of 0.986 for Hypertrophy.

**Figure 4:**
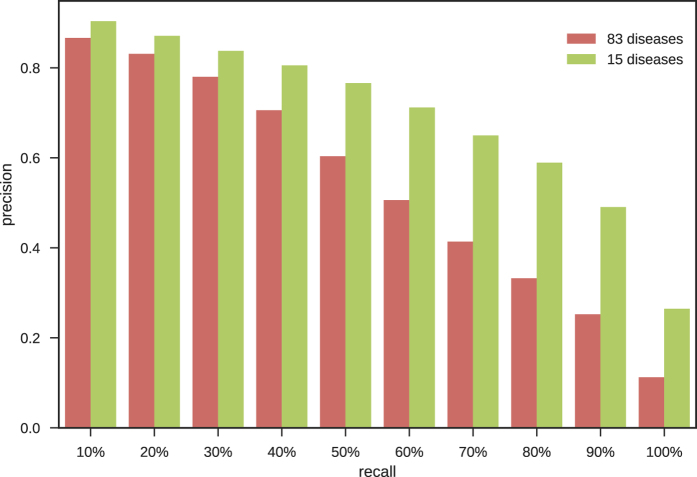
Average precision obtained for 10 different level of recall using 15 or 83 diseases.

The precisions obtained at several levels of recall for the set of 83 diseases are given in Fig. 4 (red bars). The precision decreases for higher level of recall. It is just below 0.8 (0.7802) for a recall rate of 30%. The R-precision measure is a way to obtain comparable results when the number of true associations is very different (as this is the case in this work). It is defined as the precision obtained for the top R results, with R equals to the number of microRNAs associated with the disease^30^. For the 83 diseases, the R-precision of our method is equals to 0.625.

### Comparison with other methods

A majority of diseases are associated with few miRNAs. In the literature, the performances of miRNA-disease association methods were often based on a set of 15 diseases that are associated with the largest number of miRNAs.

We compared our method with our previous manually configured version of MiRAI^10^ and 6 other methods applied on the same set of 15 human diseases associated with numerous miRNAs (Table 3). The AUC obtained by our tool range from 0.825 to 0.926 with an average AUC value of 0.880. The performance is slightly better than other methods and our previous manually configured version of MiRAI (AUC scores ranging from 0.796 to 0.928 with an average of 0.867).

**Table 3:** Prediction results for diseases associated with the largest number of miRNAs.

Disease name	RWRMDA^74^ 2012	Chen *et al*.^75^ 2013	HDMP^76^ 2013	RLSMDA^77^ 2014	MIDP^78^ 2015	Liu *et al*.^4^ 2016	MiRAI^10^ 2016	MiRAI + EA
								2017
Acute myeloid leukemia	0.839	0.716	0.858	0.853	0.913	0.871	0.895	0.906
Breast neoplasms	0.785	0.653	0.801	0.832	0.838	0.826	0.864	0.858
Colorectal neoplasms	0.793	0.662	0.802	0.831	0.845	0.833	0.864	0.868
Glioblastoma	0.68	0.607	0.7	0.714	0.786	0.839	0.898	0.872
Heart failure	0.722	0.761	0.77	0.738	0.821	0.812	0.796	0.847
Liver carcinoma	0.749	0.613	0.759	0.794	0.807	0.802	0.808	0.825
Lung neoplasms	0.827	0.606	0.835	0.855	0.876	0.925	0.904	0.926
Melanoma	0.784	0.642	0.79	0.807	0.837	0.834	0.849	0.875
Ovarian neoplasms	0.882	0.644	0.884	0.909	0.923	0.896	0.874	0.906
Pancreatic neoplasms	0.871	0.684	0.895	0.887	0.945	0.901	0.928	0.925
Prostatic neoplasms	0.823	0.629	0.854	0.841	0.882	0.842	0.871	0.872
Renal cell carcinoma	0.815	0.627	0.833	0.839	0.862	0.815	0.869	0.882
Squamous carcinoma	0.819	0.676	0.82	0.849	0.87	0.872	0.883	0.888
Stomach neoplasms	0.779	0.628	0.787	0.797	0.821	0.798	0.815	0.848
Bladder neoplasms	0.821	0.632	0.85	0.845	0.897	0.851	0.884	0.900
**AVERAGE AUC**	**0**.**800**	**0**.**652**	**0**.**816**	**0**.**826**	**0**.**862**	**0**.**848**	**0**.**867**	**0**.**880**

The AUC scores of MiRAI configured with an evolutionary algorithm (MiRAI + EA) are compared with the scores of manually configured MiRAI and 6 other methods.

The improvement brought by the tuning of MiRAI using an EA is more significative if we compare the results obtained for all 83 diseases, since the average AUC jumps from 0.867 to 0.897 (Supplementary Table S1). This result is not surprising because the EA has been designed with the goal of maximizing this value.

The precisions obtained at several levels of recall for the set of 15 diseases are given in Fig. 4 (green bars). At 10% recall, the precision is 0.903. It is still above 0.8 (0.805) at 40% recall meaning that the method retrieves a significant proportion of associations with a good precision. The R-precision measured for the 15 diseases associated with numerous miRNAs is 0.691.

The performance of our method cannot be easily compared with methods that are able to perform predictions with few or even no annotation. However, we can state that MiRAI, even optimized with EA, is outclassed by recent methods such as NCPMDA^7^ that exhibits an AUC score of 0.9173 (althought calculated with leave-one-out cross validation and not a 5-fold validation), or PBMDA^8^ with an AUC score of 0.9172. Our method is also clearly far behind ensemble-based method combining the predictions of multiple algorithms that reach an impressive AUC score of 0.9226.

Our method nevertheless obtains good results. It is not capable of performing predictions with few existing annotations but it allows to highlight potential false associations contained in the miRNA-disease association databases.

### Detection and correction of mis-annotations

Identifying miRNAs and diseases whose associations are reported in HMDD and that are represented by very distant vectors in the vector space (indicating dissimilarities) allows to highlight putative false associations. With our method, we identified 86 associations with a significant score of invalidation (Supplementary Table S2). A manual step of confirmation was undergone by checking the associated publications of each association. The associations miRNA/disease used from the reference database must correspond to the comparison of miRNA expression between healthy and sick cases in Human. Among associations highlighted by the invalidation process of MiRAI, more than half (57%) comes from works that study the effect of molecules or treatments on cancer cells^31–33^ or the evolution of miRNA expression in the cancer progression^34, 35^. 19% of infirmed associations are linked with research based on circulating miRNAs. Although miRNAs contained in serum or blood represent tremendous potential biomarkers, they are currently contested by a part of the scientific community^36^. Pending further results, we have preferred to omit the data from works on circulating miRNAs. The rest of invalidations corresponds to control problems (like the use of control cells from a sick patient)^37, 38^, the automated retrieval of data from tables without taking account of statistical scores^39^ and bioinformatics predictions^40^. Only 8 infirmed associations could not be explained by the reading of publications^41–47^. One association could not be checked because the originated publication is entirely in Chinese^48^.

After the manual-checking step, confirmed invalidations were removed from the reference database to increase the prediction success.

### Evaluation of predictions quality

We executed a version of MiRAI tuned with the parameters given by the evolutionary algorithm on the updated list of miRNA-disease associations.

For the 15 important diseases listed in Table 3, we obtained an average AUC value of 0.889, which is a significative improvement compared to the scores obtained with HMDD (Supplementary Table S3). The improvement was, however, less by considering all diseases. Now, the average score is 0.898, compared to 0.897 previously obtained (Supplementary Table S4). These are nevertheless excellent scores that characterize a very good classifier.

### mir-188 and mir-765 are predicted to play a role in several diseases

From the revised reference database, MiRAI found 126 potential new associations between miRNAs and diseases (Supplementary Table S5). Since HMDD is upgraded manually, we performed a manual-checking step of these associations in order to control if a recent publication was already released and explains the results. 54 of our predictions were indeed discovered in the last two years (confirmed associations are highlighted in green in Supplementary Table S5).

Among the 72 remaining putative associations, most of them imply mir-188 and mir-765 with several diseases (32 and 21 respectively) (Supplementary Table S6).

**Figure 5:**
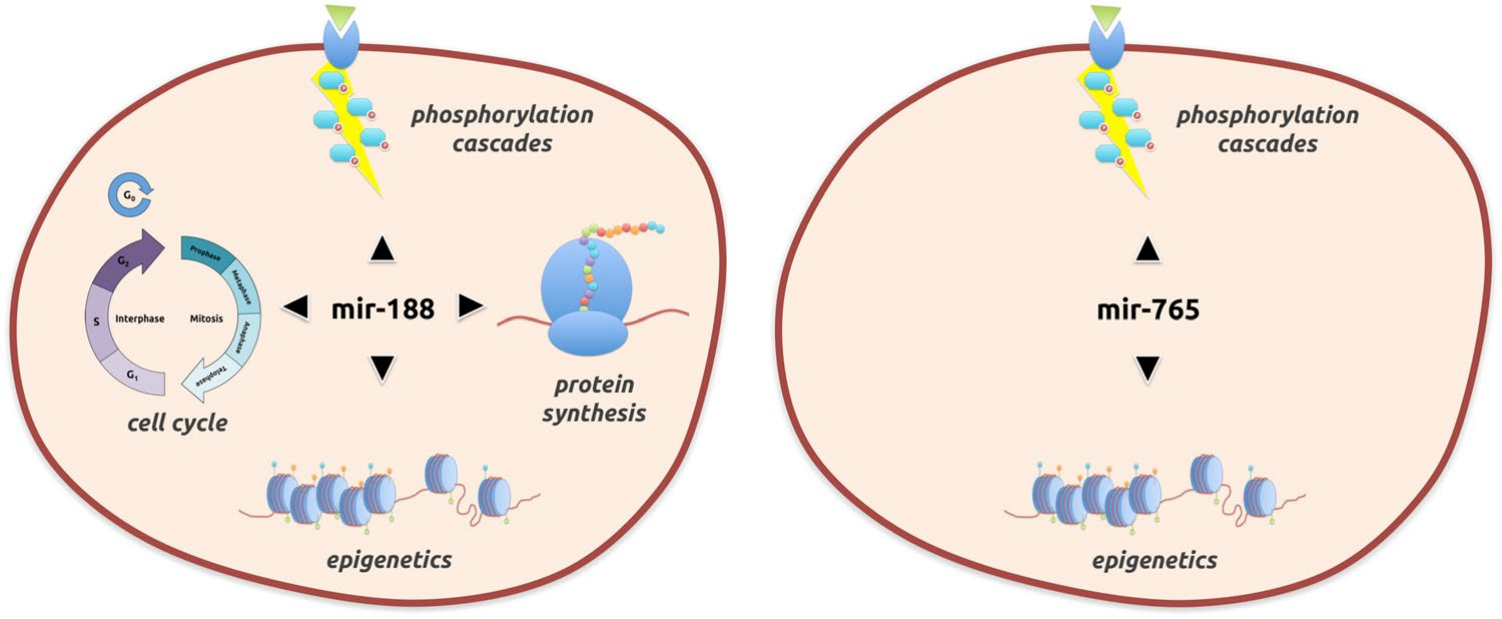
Main biological processes and pathways known for mir-188 and mir-765.

Mir-188 is a 21-nucleotide element located in the human genome on the X chromosome (50003503–50003588 +). It was first isolated in 2003 from kidney of mice by Tuschl’s team in Germany^49^. Mir-188 dysregulation is reported in various diseases such as Alzheimer’s disease^50, 51^, azoospermia^52^, breast cancer^53^, some carcinoma^54–56^, leukemia^57, 58^, infarction^59^, myeloma^60^, pre-eclampsia^61^ and prostate cancer^62^. Mir-188 takes part in the cell cycle maintenance (cell proliferation, G1/S cell cycle transition, tumor colony formation) by targeting genes implied in the cell cycle checkpoints (CCND1, CCND3, CCNE1, CCNA2, CDK4 and CDK2)^63^. Its role was also shown in cellular epigenetic processes^52^ (histone code) and in protein synthesis activation^63^. Our analysis reveals its potential implications in at least 32 other diseases, including cardiovascular diseases, neural disorders and autoimmune diseases. Its action on key fundamental cell functions may explain its potential implication in such a large range of diseases (Fig. 5).

Mir-765 is a 21-nucleotide element located in the human genome on the first chromosome (156936131–156936244 −). It was first isolated in 2006 from human embryonic and primary cells by Cuppen’s team in The Netherlands^64^. mir-765 is reported inhibiting the phosphorylation of eNOS^65^ and ERK/Akt/AMPK signalling by targeting the apelin^66^, an endogenous ligand of G-protein. Mir-765 also interferes with the MAPK pathway by repressing the neurotrophic receptor tyrosine kinase^67^. These pathways are involved in the regulation of cell cycle induced by an external stimulus (blocking of cell-cycle progression at the G2/M transition, cell migration and invasion). Mir-765 decreases the level of HMGA1^68^, a non-histone chromatin protein involved in the regulation of DNA-dependent 3R processes (replication, recombination and repair). It is implied in many diseases: oligoasthenozoospermia^69^, hepatocellular carcinoma^70^, failing heart^71^ and breast^72^, prostate^68^ and rectal cancers^73^. Our predictions report a potential link of mir-765 with 21 diseases, including neural disorders, cardiovascular diseases, rheumatoid, various lymphoma and carcinoma, leukaemia, liver cirrhosis and female reproductive system cancers (Fig. 5).

## Conclusion and Perspectives

The cancer is a multi-step disease. The accumulation of mutational events on DNA during a life may conduct to the emergence of a cancer. These events are sorted in two categories according to their origins: environmental (e.g. cigarette smoke, radioactivity, alcohol, etc.) and intrinsic (e.g. DNA replication error, genetic heritage, reactive oxygen species, etc.). Statistically, we will develop at least 3 polyps (benign gut cancers) in our human life.

In the 1990s, scientists discovered the presence of small genetic elements in DNA samples of worms and called them micro-RNA. For many years, these elements have ended up in the bottom of electrophoresis tanks without ever being analyzed and yet researchers showed their implication in various metabolic pathways (gene regulation, epigenetics, mitosis, etc.) as well as in diseases (cancers, Alzheimer’s syndrome, heart failure, etc.).

We designed MiRAI, a method based on distributional semantics to predict associations between miRNAs and diseases. Parameters of MiRAI were tuned using an evolutionary algorithm and the performances of the method were increased by 32%. Application of MiRAI on HMDD data highlighted potential new associations between miRNAs and diseases. Among them, mir-188 and mir-765 present the most of new predictions with diseases, and could be ubiquitous biomarkers of some diseases. These two miRNA are known to be linked with several main biological processes and pathways such as cell cycle or epigenetics. More investigations in laboratories are needed to confirm these hypotheses. MiRAI has also been used to highlight potential false associations contained in the miRNA-disease association databases.

The increase of works on miRNAs leads to the development of microRNA-based biotechnology mainly for human health purposes. Two major axes are currently conducted: one on the early diagnosis and the other on the gene therapy.

Most of publications referring to miRNA present observations of miRNA expression between healthy and sick cases. miRNA deregulations are shown in various diseases such as cancers or neural disorders. miRNA patterns appears to be interesting biomarkers of syndromes or their evolution/aggravation. The holy grail of this development is to use signatures of circulating miRNAs from blood or lymph samples, which are less invasive than tissue biopsies.

Several miRNAs were identified as tumor suppressor or playing key roles in sickness. Among them, some have an expression level reduced or lost in virtually. A supply with miRNA mimics in cells could prevent or cure these diseases. Moreover, unlike current cancer treatments that focus on one or two oncogenes, miRNAs generally target several genes and could be a generic solution to handle several public health problems. A huge R&D effort on drug delivery methods is, however, yet to achieve.

MiRAI fits perfectly into this dynamic of innovation by carrying concrete solutions to R&D purposes of this domain:

1. Increase the chances to discover new associations;
2. Help scientists to focus on targets with strong potentials for success;
3. And quantify the coherence of a discovery according all available data.

Moreover, with the rise of data on miRNA and dedicated databases, the efficiency and the accuracy of prediction solutions like MiRAI will continue to improve year after year.

## Electronic supplementary material

Supplementary  Information

Supplementary Table S1

Supplementary Table S2

Supplementary Table S3

Supplementary Table S4

Supplementary Table S5

Supplementary Table S6
