# Supplementary material for: Prediction of miRNA-disease Associations using an Evolutionary Tuned Latent Semantic Analysis
Source: Sci Rep. 2017 Sep 5;7:10548. doi: 10.1038/s41598-017-10065-y (PMC5585369; doi:10.1038/s41598-017-10065-y)
Supplement: Supplementary file 1 — Supplementary Information [file 41598_2017_10065_MOESM1_ESM.pdf]

Prediction of miRNA-disease Associations using an Evolutionary Tuned Latent Semantic Analysis

Authors: Denis Pallez[1+], Julien Gardès2[+], Claude Pasquier[1+]*

Affiliations:

[1]Université Côte d'Azur, CNRS, I3S, France.

[2]BIOMANDA, 2720 Chemin St Bernard, Les Moulins I Batiment 4, 06220 Vallauris, France

*Correspondence to: claude.pasquier@unice.fr.

Supplementary Materials

Supplementary Figure S1: Best, average and worst population fitness evolution for each experiment using PGPABDE.

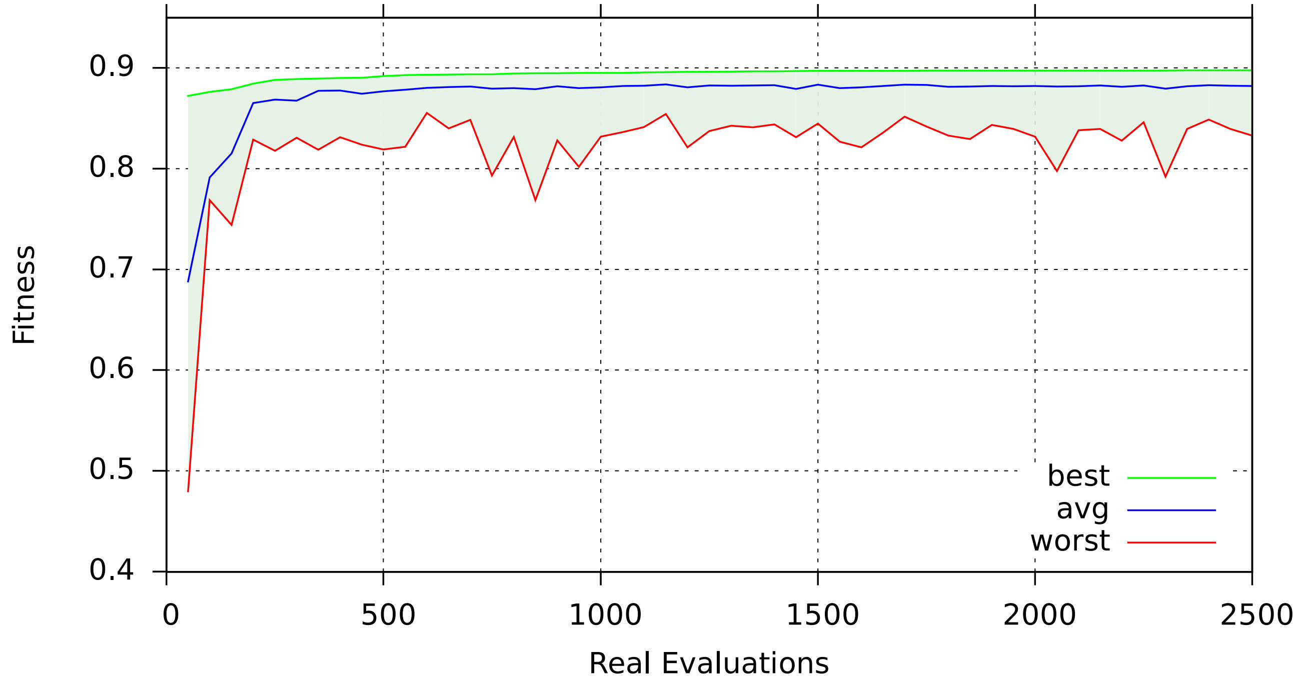

Supplementary Figure S2: Best, average and worst population fitness evolution for each experiment using PBDE.

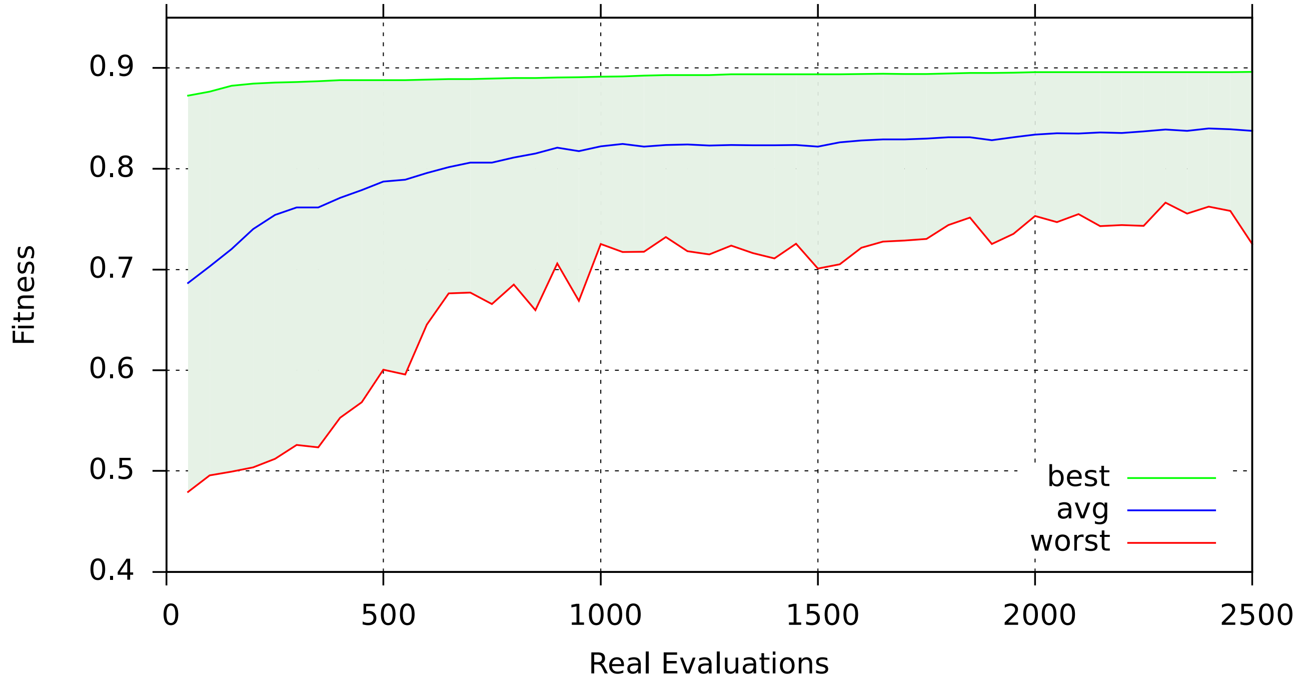

Other supplementary datasets stored in separate files.

Supplementary Table S1: Results obtained by EA tuned MiRAI for 83 diseases.

Supplementary Table S2: List of putative false association contained in HMDD.

Supplementary Table S3: AUC scores obtained for 15 important diseases using an updated list of miRNA-disease associations..

Supplementary Table S4: AUC scores obtained for 83 diseases using an updated list of miRNA-disease associations.

Supplementary Table S5: List of potential new associations identified by EA tuned MiRAI.

Supplementary Table S6: List of unconfirmed new associations.
